# Effects of caffeine on locomotor activity in streptozotocin-induced diabetic rats

**Published:** 2016

**Authors:** SV Bădescu, CP Tătaru, L Kobylinska, EL Georgescu, DM Zahiu, AM Zăgrean, L Zăgrean

**Affiliations:** * Department of Physiology and Fundamental Neuroscience, “Carol Davila” University of Medicine and Pharmacy, Bucharest, Romania; ** Department of Ophthalmology, “Carol Davila” University of Medicine and Pharmacy, Bucharest, Romania; Emergency Eye Hospital, Bucharest, Romania

**Keywords:** diabetes mellitus, caffeine, locomotor activity, streptozotocin, rat

## Abstract

Diabetes mellitus modifies the expression of adenosine receptors in the brain. Caffeine acts as an antagonist of A1 and A2A adenosine receptors and was shown to have a dose-dependent biphasic effect on locomotion in mice. The present study investigated the link between diabetes and locomotor activity in an animal model of streptozotocin-induced diabetes, and the effects of a low-medium dose of caffeine in this relation. The locomotor activity was investigated by using Open Field Test at 6 weeks after diabetes induction and after 2 more weeks of chronic caffeine administration. Diabetes decreased locomotor activity (total distance moved and mobility time). Chronic caffeine exposure impaired the locomotor activity in control rats, but not in diabetic rats. Our data suggested that the medium doses of caffeine might block the A2A receptors, shown to have an increased density in the brain of diabetic rats, and improve or at least maintain the locomotor activity, offering a neuroprotective support in diabetic rats.

**Abbreviations**:

STZ = streptozotocin, OFT = Open Field Test

## Introduction

Diabetes is one of the most common diseases of the 21st century with a rapidly increasing incidence in the next decade that affects people worldwide; presently one in eleven adults is considered to be diabetic [**1**]. Diabetes mellitus manifests with hyperglycemias due to the lack of insulin secretion or defects of insulin action upon its receptors, or both. Along with the hyperglycaemic state, diabetes was found to produce structural changes in the brain [**2**] and patients present a variety of neurological consequences such as peripheral neuropathy and a higher risk for dementia [**3**]. In patients with Parkinson disease, diabetes induces a worsening of the pathology [**4**] and may be involved in postural instability and gait difficulty [**5**]. Also, diabetes exacerbates brain atrophy and decline of cognitive functions in these patients [**6**].

The need for developing new strategies in combating locomotor disabilities, brought into attention caffeine, the most often used psychoactive substance worldwide. Caffeine acts by blocking adenosine A1 and A2A receptors. Adenosine is involved in locomotor processes [**7**], A1 receptors are colocalized with dopamine D1 receptors, and A2A receptors are colocalized and communicate with D2 receptors [**8**]. Caffeine might be of therapeutic help because of its ability to interact with dopamine receptors and modify signal transduction in the striatal neurons. Also, it was suggested that it acts as a neuroprotective agent by balancing the effects of dopaminergic neural loss [**9**]. However, caffeine administration has a biphasic effect: in low doses, it increases locomotor activity [**7**], while high doses impair locomotion in rodents [**10**].

The present study investigated the link between diabetes mellitus and locomotor activity in an animal model of streptozotocin-induced diabetes and the possible effects of caffeine in this relation.

## Materials and methods

Experiments were performed by using male Wistar albino rats, weighing 300-350g at the beginning of the experiments, aged 3-4 months old. All the animals were kept in standard illuminating conditions (12 hours day cycle), in transparent cages, at constant temperature (22° ± 2°C), with free access to food and water. The animals were provided by “Carol Davila” University of Medicine and Pharmacy, Bucharest, Romania. All the animal procedures were carried out with the approval of the local ethics committee in accordance with the European Communities Council Directive 86/609/EEC on the protection of animals used for scientific purposes.

**Induction of Diabetes**

One of the animal models of diabetes is obtained through streptozotocin administration and its subsequent destruction of pancreatic beta cells that secret insulin [**11**], mimicking a type 1 diabetes. Diabetes was induced by a single intraperitoneal injection of 50 mg/ kg BW streptozotocin (STZ) diluted in 0.9% saline solution. Aged-matched control rats received an equivalent of 0.9% saline solution. Blood samples from fasting rats were taken from the tail vein after one week of STZ or vehicle injection. Rats with fasting glycaemia over 250 mg/ dl were considered diabetic and were included in the study. Rats with glycaemia over 600 mg/ dl were eliminated from the study due to poor biological potential that would not ensure long-term survival. In the end, 19 animals were included: 9 in the control group and 10 in the diabetic group.

**Caffeine administration**

Caffeine (1,3,7 - trimethylxanthine) was administered orally for 2 weeks in the water (20 mg/kg/d), starting the next day after the last behavioral test in diabetic and control rats, and then continued throughout the experiments to diabetic (N=5) and control (N=5) rats.

**Open field test**

Six weeks after the STZ injection, the locomotor activity of the rats was assessed with Open Field Test (OFT). All the tests took place in the same time interval, between 8:00-12:00 and 14:00-17:00, in order to reduce circadian rhythm effects. All the behavioral tests were repeated after 2 weeks of continuous caffeine administration.

To perform OFT, the rat was placed for 10 minutes in an unfamiliar arena of 40 x 40 cm with walls of 40 cm height. The locomotor activity was evaluated by measuring the total distance moved (cm), mobility time (s), speed (cm/ s), time spent in the central area of the arena (an area virtually delimited at 10 cm from the walls) and number of center entries. The arena’s surfaces were black in order to allow automatic identification of the rat by white-black contrast. 

Noldus Ethovision XT 5 video tracking system was used to record and automatically analyze the behavioral tests.

## Results

The results were analyzed by using SPSS 22.0. The distribution of the data was checked for normality by using the Shapiro-Wilk test and parametrical test (T-test) for continuous variables with Shapiro-Wilk p>0.05, otherwise, non-parametrical tests (Mann-Whitney-U, Kruskall-Wallis, Wilcoxon’s Signed Ranks Test, Spearman’s correlation coefficient) were used. The glycemic values were reported as mean ± sem.

After 1 week from the STZ injection, blood glucose levels increased with a medium glycemic value of 455.50 ± 90.30 mg/ dl. After 6 weeks from the STZ injection, prior to OFT, diabetic rats had a medium glycemic value of 517.40 ± 76.96 mg/ dl. Caffeine administration did not modify the glycemic values after two weeks of chronic administration: median glycaemia in caffeine treated diabetic rats was 523.30 ± 75.71. Glycemic values of control rats did not differ across the study, as the median glycaemia was 113.60 ± 8.77 mg/ dl after 1 week from vehicle injection, 102 ± 5.63 mg/ dl after 6 weeks and 99.60 ± 7.65 mg/ dl after 2 weeks of caffeine administration.

**Fig. 1:**
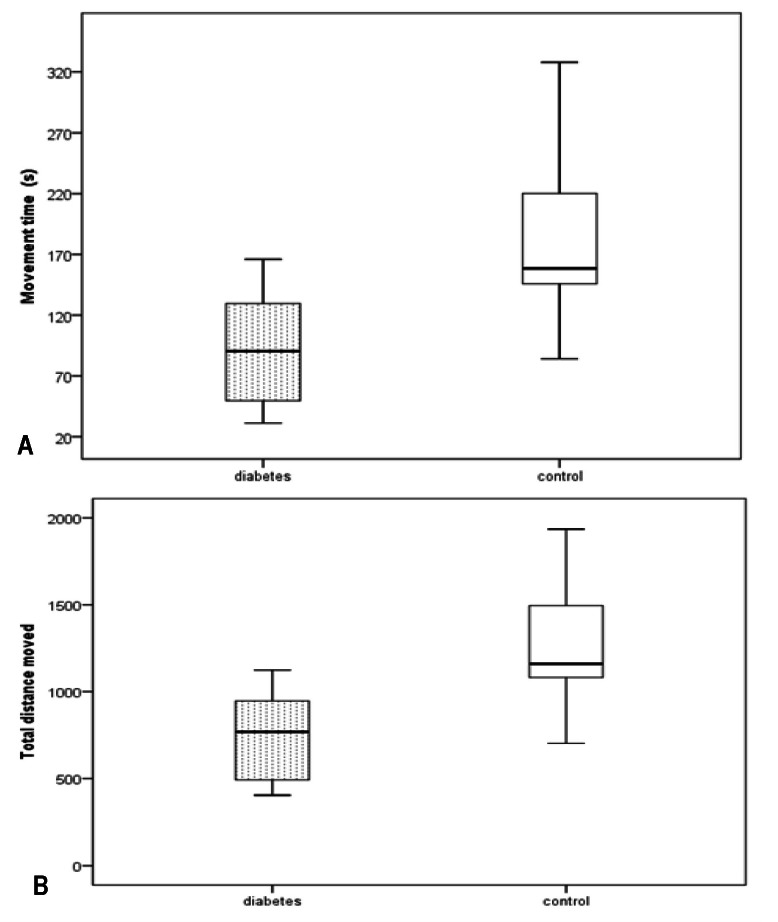
Boxplot of the total mobility time in each group at the OFT, **Fig. 1B** Boxplot of the total distance moved in each group at the OFT. The rats in the control group spent more time being mobile (n1 = 9, n2 = 10, average control group mobility time = 179.964 ± 78.208s, average diabetic group mobility time = 93.904 ± 45.293s, t(17) = 2.975, p = 0.008, 95%CI = [25.023, 147.097s]) and they moved more (n1 = 9, n2 = 10, total distance control group = 1244.114 ± 423.863 cm, total distance diabetic group = 734.962 ± 264.396 cm, t(17) = 3.122, p = 0.006, 95%CI = [162.17, 838.12cm])

At the Open Field Test, total distance moved, mobility time, center time, and number of center entries, were compared. There was a significant reduction in the time spent moving and the total distance moved between the diabetic group and the control group rats (**Fig. 1A, 1B**). 

There were no differences between the number of center entries (Z = 53.5, n1 = 9, n2 = 10, p = 0.497) or in the time spent in the center of the OFT arena (Z = 50.5, n1 = 9, n2 = 10, p = 0.661).

**Fig. 2:**
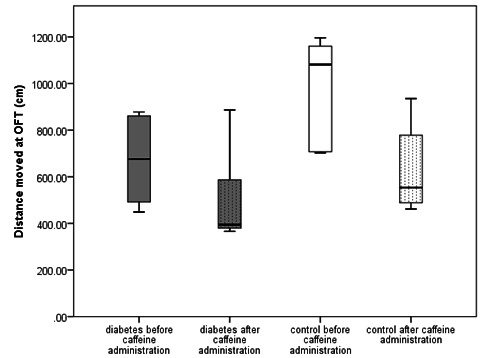
Boxplot of the total distance moved (cm) at the OFT in each group before and after caffeine administration. The rats in the control group moved less after caffeine administration than before (Z = -1.753, n = 5, p = 0.04 (one-tailed), median distance before caffeine administration = 1081.21, IQR = 472.81, median distance after caffeine administration = 553.42, IQR = 381.72), while movement in diabetic rats did not change (Z = -0.944, n = 5, p = 0.19, median distance before caffeine administration = 675.72, IQR = 398.76, median distance after caffeine administration = .393.99, IQR = 364.15)

After caffeine administration, the rats in the control group were less mobile than before (Z = -2.023, n = 5, p = 0.043, median movement time before caffeine = 156.48s, IQR = 67.16s, median movement time after caffeine = 62.8s, IQR = 60.56s), with a consecutive decrease in the total distance moved (**Fig. 2**). There were no differences in the measured parameters in the diabetic group before and after caffeine administration. However, after caffeine administration, there were no longer differences regarding the mobility and the total moved distance between the control and the diabetic group (**Fig. 2**).

## Discussion

In the present study, the locomotor activity in diabetic adult Wistar rats and the influence of chronic caffeine treatment in moderate dose were investigated. Our results showed that diabetes decreased locomotor activity and chronic caffeine exposure impaired locomotor activity in control rats, but not in diabetic rats.

The evaluation of locomotor activity is often performed by using open field-testing [**10**,**12**]. While most experiments on rodents use acute administration of caffeine, our study used a chronic caffeine treatment in order to get closer to the human consumption model. Humans consume caffeine chronically and its intake is divided up during the day [**13**]. Due to differences in caffeine metabolism between rats and humans, a dose of 10 mg/ kg BW in rats is comparable with approximately 250 mg of caffeine in a 70 kg human being, representing 2 to 3 cups of coffee [**13**]. In our study, we used a moderate dose of caffeine intake, comparable with human consumption, measured by Frary et al. to be between 160 and 336 mg/ day in United States adults [**14**].

Studies on locomotor activity after caffeine administration found a biphasic effect according to the amount of caffeine doses. After an acute administration of low to medium doses, caffeine appears to increase locomotor activity in adult rodents [**7**], while after acute high doses; the activity is decreased [**10**]. Moreover, Marin et al. showed that there are differences produced not only by the dose of caffeine used, but also by that adult and adolescent Wistar rats respond differently to caffeine [**15**]. In the present study, the chronically administered medium dose of caffeine produced an inhibitory effect upon the locomotor activity of the control rats, comparable to high caffeine doses in acute administration [**10**].

Caffeine primary acts as an antagonist of adenosine A1 and A2A receptors, as these receptors bind caffeine at low concentrations [**13**]. The stimulant action of caffeine was related to its effect on A2A receptors [**7**], as A2A receptor knockout mice showed a depressed locomotor activity [**16**]. The depressed locomotor activity induced by caffeine was suggested to be a consequence of A1 receptor antagonism [**16**]: A1 receptors might be blocked by high doses of caffeine or they might counteract the stimulant effect induced by the blockade of A2A receptors. However, after studying mice lacking A1 or/ and A2A receptors, Halldner et al. considered that the inhibitory effect of high doses of caffeine is independent of the A1 and A2A receptors [**17**]. While A2A receptors are found especially in the striatum [**18**], a dopamine rich region of the brain, A1 receptors are abundant in different brain regions, mainly in the hippocampus, cortex, but also in the basal ganglia and cerebellum [**19**]. Studies in mice showed that chronic caffeine ingestion caused a significant increase of A1 adenosine receptors in cerebral cortical neurons, while the density of A2A receptors remained unaltered [**20**,**21**]. The increase of A1 receptors might explain the decreased locomotion observed in the control rats after chronic caffeine administration.

In accordance with other studies, we found an impaired locomotor activity in diabetic rats [**22**]. However, after caffeine administration, the locomotor activity decreased only in control rats, but not in the diabetic ones, suggesting there might be some receptor changes in the diabetic brain. Chronic hyperglycemias of the streptozotocin-induced diabetic rats alter hippocampal metabolism [**23**] with a consecutive increase in adenosine A2 receptors in the hippocampus of diabetic rats and a down regulation of adenosine A1 receptors [**24**]. Nevertheless, we found no studies regarding possible modifications of the adenosine receptors in the striatum in diabetic rodents. Studies of chronic noxious brain conditions found the balance of A1 and A2A receptors to be altered in the brain, with stressful stimuli increasing A1 receptors density, while decreasing A2A receptors density [**25**]. Considering the adaptive changes of adenosine receptors in the hippocampus of diabetic rats and from the premises of imbalance produced by stressful stimuli in the whole brain, we might extend these results to streptozotocin-induced diabetes and offer a possible explanation for the observed locomotor activity. The decrease of A1 receptors in diabetic rats might not be balanced by their increase after chronic caffeine administration, while the density of A2A receptors remain increased in diabetic condition and unaltered after chronic caffeine exposure.

## Conclusion

Diabetes leads to modifications of morphology and function of the central nervous system, decreasing mobility and locomotor activity. Low – medium doses of caffeine improve or at least maintain the locomotor activity, offering a neuroprotective support in diabetic rats. In the light of previous research, our results suggest that caffeine might block the increased number of A2A receptors in different diabetic brain regions. Further studies should be performed on different doses of caffeine in diabetic rats in order to see if the biphasic effects of caffeine observed in the control rats also appear in the diabetic ones. Evaluating the alteration in the balance of A1 and A2A receptors in diabetic rats and assessing the caffeine effects on this balance are the next steps for the better understanding of diabetic brain and caffeine-diabetes interactions.

**Sources of funding **

The study was supported by “Carol Davila” University of Medicine and Pharmacy through “Young Researchers” Grant no. 28482/2012.

**Disclosures**

None
